# Terminalia catappa aqueous leaf extract reverses insulin resistance, improves glucose transport and activates PI3K/AKT signalling in high fat/streptozotocin-induced diabetic rats

**DOI:** 10.1038/s41598-022-15114-9

**Published:** 2022-06-23

**Authors:** Franklyn Nonso Iheagwam, Olawumi Toyin Iheagwam, Michael Kemjika Onuoha, Olubanke Olujoke Ogunlana, Shalom Nwodo Chinedu

**Affiliations:** 1 Department of Biochemistry, Covenant University, P.M.B. 1023, Ota, Ogun State Nigeria; 2 Covenant University Public Health and Wellbeing Research Cluster (CUPHWERC), Covenant University, P.M.B. 1023, Ota, Ogun State Nigeria; 3 Geniebook Associates, Ikeja, Lagos State Nigeria; 4 Covenant University Health Centre, Covenant University, P.M.B. 1023, Ota, Ogun State Nigeria

**Keywords:** Mechanism of action, Natural products, Pharmacology, Chemical biology

## Abstract

Rising prevalence of type 2 diabetes mellitus (T2DM) in sub-Saharan Africa has necessitated surveys of antidiabetic medicinal plants. This study assessed the antidiabetic mechanism of *Terminalia catappa* aqueous leaf extract (TCA) in high fat/low dose streptozotocin-induced type 2 diabetic rats. T2DM was induced by a combination of high-fat diet and low dose STZ (30 mg/kg bw) and the animals were administered with TCA (400 and 800 mg/kg bw) orally daily for 28 days. Biochemical parameters and indices for diabetes including renal function tests and pancreatic histology were evaluated. Relative expression of hepatic insulin resistance, signalling and glucose transport genes were also assessed. Induction of T2DM resulted in significant (*p* < 0.05) weight loss, dysregulated glucose level and clearance, electrolyte imbalance and disrupted diabetic biochemical parameters. Diabetes onset also perturbed β-cell function and insulin resistance indices, damaged pancreas microanatomy, while disrupting the expression of insulin receptor substrate 1 (*IRS-1*), phosphatidylinositol 3-kinase (*PI3K*), protein kinase B (*AKT*) and glucose transporter isoform 4 (*GLUT-4*) mRNA. Oral treatment of diabetic animals with TCA significantly (*p* < 0.05) ameliorated alterations due to T2DM induction in a manner comparable with glibenclamide. These results suggest TCA exerts its antidiabetic action by reversing insulin resistance, improving glucose transport and activating PI3K/AKT signalling.

## Introduction

Diabetes mellitus (DM) is a metabolic disease with characteristic high plasma glucose concentration due to either insulin deficiency and/or impaired insulin activity. Insulin release and action are affected by numerous factors such as lifestyle activities, genetics and epigenetics predisposition^1^. About 6.7 million and 416,000 deaths worldwide and Africa respectively, were associated with DM, ranking it among 90th percentile of diseases that cause death in year 2021^2^. Type 2 diabetes mellitus (T2DM) is the most predominant type of DM, which has constituted a public health challenge by contributing to poor quality of life, morbidity and mortality^3^. The prevalence and incidence of T2DM in Nigeria and sub-Sahara Africa are increasing at an alarming rate requiring an immediate solution^4,5^. Weight loss, polyuria, polydipsia, and hyperglycaemia are the common symptoms associated with T2DM. The β-cells of the pancreas are most affected during the onset of the disease, leading to dysregulated blood glucose levels and uptake with aberrant insulin secretion and action^6^.

Signal transduction of insulin (IST) is a mechanistic pathway involving mediators and enzymes that facilitate glucose entry into tissues and organs via glucose transporter type 4 (GLUT-4)^7,8^. The binding of insulin to the α chain of its receptors (IRs) initiates IST by inducing structural changes in the β chain via autophosphorylation at three tyrosine residues^9^. Hence activating its autokinase activity, leading to the phosphorylation and recruitment of different adaptor proteins (insulin receptor substrates (IRS), Shc protein and adapter protein (APS) with a pleckstrin homology (PH) and Src homology 2 (SH2) domains to provide an appropriate binding site for the IRS-1 phosphorylation, activation and downstream processing which involves phosphoinositide 3-kinase (PI3K) and protein kinase B (Akt) activation facilitating glucose entry into cells by glucose transporter isoform 4 (GLUT-4) localization^10,11^. Defects in these steps, enzymes or mediators involved in the normal functioning of IST can potentially impair the process contributing to T2DM and insulin resistance^7,12^.

Medications that have been used over time in the treatment of DM, especially T2DM, have plants and microbes as their source (such as galegine, pycnogenol and phenolics compounds from plants while miglitol, acarbose and voglibose from microbes)^13^. Plants contain phytoactive compounds and secondary plant metabolites that exert antidiabetic effects either singly or synergistically^14^. Upregulating GLUT, insulinomimetic effects, insulinotropic effects, modification of glycogen metabolism, incretin mimetic and incretin enhancement to alter glucose metabolism/glucose homeostasis are significant mechanisms exhibited by medicinal plants^15^. *Terminalia cattapa*, also known as tropical almond, is a well known medicinal plant in Nigeria with antimicrobial^16^, antibacterial^17^, anti-inflammatory, anti-HIV^18^, hypoglycaemic^19^, analgesic, wound healing, antioxidant, radical scavenging, hepatoprotective, anticancer, antimutagenic and antiaging properties^20^. Aqueous leaf extract of *T. cattapa* has been reported to palliate redox imbalance and inflammation in high fat/low dose streptozotocin-induced diabetic rats^21^. Previous reports have shown elevated oxidative stress and increased free radical generation truncate IST normal processes, leading to unfavourable downstream effects^11,22^. *T. cattapa* might ameliorate the disruption of IST in high fat/low dose streptozotocin-induced diabetic rats to exacerbate the onset of DM and insulin resistance. In addition, *T. catappa* aqueous formulation is used in folklore to treat hyperglycaemia and diabetes by different ethnic groups in Nigeria^23,24^. Nonetheless, there is a paucity of information with respect to type 2 antidiabetic molecular mechanism of action. Hence, this study assessed the antidiabetic mechanism of *Terminalia catappa* aqueous leaf extract (TCA) in high fat-low dose streptozotocin-induced type 2 diabetic rats with regards to insulin resistance, signalling and glucose transport.

## Materials and methods

### Chemicals and reagents

The acquired RT-PCR kit (TransGen EasyScript) was from TransGen Biotech Co. Ltd (Beijing, China). Diagnostic and ELISA kits were bought from Randox Diagnostics (Ireland, UK), Teco Diagnostics (California, USA) and Hangzhou Eastbiopharm (Beijing, China), respectively. Streptozotocin (STZ) was purchased from Solarbio Science and Technology (Beijing, China), while molecular primers were from Integrated DNA Technologies (Iowa, USA). All other chemicals and reagents of analytical grade were from Sigma-Aldrich, Germany.

### Plant specimen collection, identification and preparation

*T. catappa* (TC) mature leaves were plucked from Covenant University, Ota, Nigeria. They were authenticated by Dr. J. O. Popoola and deposited at the Forestry Research Institute of Nigeria (FRIN) (FHI 112775). Crude TC aqueous leaf extract (TCA) was prepared using a rotary evaporator (Stuart RE 300/MS, Staffordshire, UK) to concentrate the aqueous filtrate as described by Iheagwam et al.^25^. The leaves were dried under shade in a well aerated room for two weeks, pulverised into a fine blend and steeped in distilled water (5% w/v) for 3 days before the aqueous filtrate was obtained. Studies complied with local and national ethical guidelines/regulations on the usage of plants.

### Experimental animals and treatment dosage determination

Thirty Wistar rats of male sex (200 ± 20 g) aged between 6 and 8 weeks were purchased from the College of Medicine, University of Lagos for this study and acclimatised for two weeks prior to the experiment. Optimal living conditions were maintained, such as a humidity of 50 ± 5%, room temperature of 23 ± 2 °C and 12 h light/dark cycle in addition to food and water provision ad libitum. Covenant University Health, Research and Ethics Committee (CHREC/031/2018) approved the experimental protocol following institution guidelines for animal care and handling documented by ARRIVE guidelines and the National Institutes of Health (NIH). A cup of *T. catappa* juice measuring about 400 mL for an adult was suggested by herbal practitioners and ethnobotnical survey first thing in the morning and last thing at night for effective management of hyperglycaemia and diabetes^23^. A weight of 4.42 g of TCA was obtained after rotary concentration of the juice (400 mL). The dosage was then extrapolated to give 391.5 mg/kg bw PO for albino rats.

### Induction of diabetes and research design

**Figure 1:**
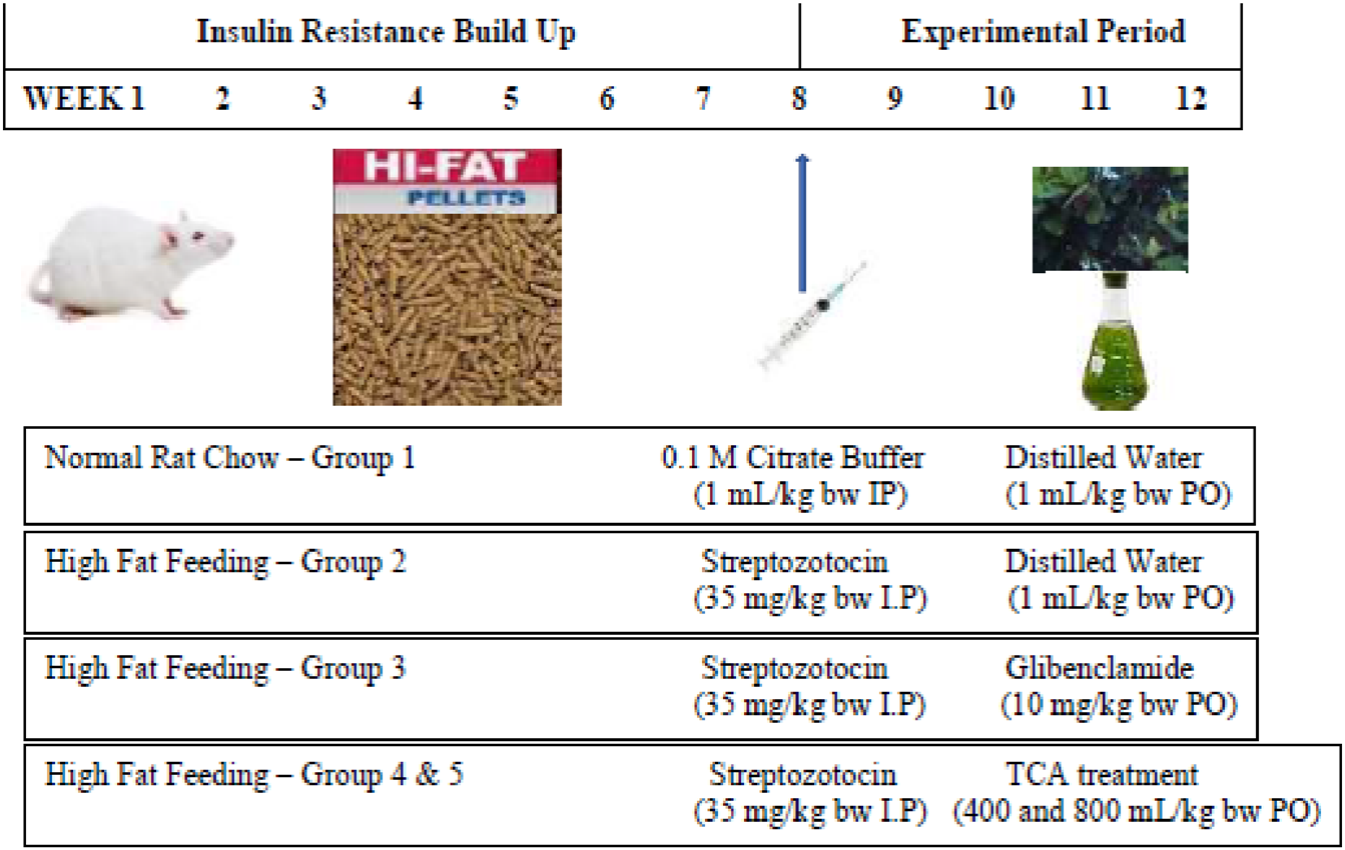
Experimental design.

The method previously described by Iheagwam, et al.^21^ was used to induce T2DM using a combination of a high-fat diet (HFD) and low dose STZ (30 mg/kg bw). Experimental animals (n = 30) were randomly placed in five (5) groups containing 6 rats each, as earlier reported in a previous study by Iheagwam et al.^21^ as depicted in Fig. 1. Treatment with TCA via oral intubation lasted for 28 days, with animal body weight and fasting blood glucose levels recorded during the experiment. The oral glucose tolerance test (OGTT) was executed on the 25th day according to the method of Irudayaraj et al.^26^. On the last day, the animals were placed on an overnight fast and thereafter placed under anaesthesia using xylazine/ketamine (5:50 g/g).

### Collection and preparation of samples

The experimental animals were sacrificed via cardiac puncture, collected blood was transferred into a heparin bottle and separated to obtain plasma and erythrocytes^27^. The liver was excised, prepared and stored as previously reported^25,28^. The excised and fat free pancreas was fixed in 10% formal saline for histology.

### Diabetic and biochemical parameter assays

The diabetic, liver and kidney function parameters were assessed in the plasma and liver. The plasma activity of α-amylase, alanine transaminase (ALT), aspartate aminotransferase (AST), alkaline phosphatase (ALP) and plasma concentration of glucose (GLUC), bilirubin (BIL), albumin (ALB), urea, creatinine (CREA), calcium (Ca), sodium (Na), bicarbonate (HCO_3_) and triglyceride (TRIG) were analysed using Randox and Teco diagnostic kits according to the manufacturers' instructions. The blood urea nitrogen (BUN) was calculated, taking into cognizance that 1 mg urea corresponds to 0.467 mg of urea nitrogen as stipulated in the manufacturers' instructions. Insulin (INS) concentration, α-amylase (AMY) and dipeptidyl peptidase IV (DPP-IV) activity in the plasma were assayed using Hangzhou Eastbiopharm ELISA kits, while protein (TP) was evaluated according to the method of Lowry et al.^29^.

Hepatic glycogen (GLYC) concentration was assessed following the method of Aba^30^ with slight modification. Briefly, 200 mg of liver tissues were homogenized in 5 mL of ice-cold KOH (30%) and boiled at 100 °C for 30 min. The homogenates were precipitated with 95% ethanol in a ratio of 1:5, five times. The residue was then resolubilized in 500 µL of water. One hundred μL each of the resolubilized samples was pipetted into tubes containing 5 mL of freshly prepared Anthrone reagent. The contents were mixed thoroughly, covered and immediately boiled for 20 min. to induce colour development. Tubes were cooled and absorbance read at 625 nm using a spectrophotometer (Thermo Scientific GENESYS™ 10S UV–Vis Spectrophotometer, USA).

### β-cell function and insulin resistance indicators

Evaluation of homeostasis model assessments for β-cell function (HOMA-β) and insulin resistance (HOMA-IR) were determined by the methods described by Mohammed et al.^31^. Quantitative insulin sensitivity check index (QUICKI), composite insulin sensitivity index (CISI) and McAuley index (MI) were calculated according to the method described by Sasidharan et al.^32^, Sahin et al.^33^ and Tsai et al.^34^ respectively. These indicators were calculated using the following formulas shown in Eqs. 1–5:

1 $$ {\text{HOMA}} -\upbeta = \frac{{\left( {20 \times fasting\,insulin} \right)}}{{\left( {fasting\,glucose - 3.5} \right)}} $$

2 $$ {\text{HOMA}} - {\text{IR}} = \frac{{ \left( {fasting\,glucose \times fasting\,insulin} \right)}}{22.5} $$

3 $$ QUICKI = \frac{1}{(\log \,insulin + \log \,glucose)} $$

4 $$ {\text{CISI}} = \frac{10 000}{{\sqrt {\left( {{\text{fasting}}\,{\text{glucose}} \times {\text{fasting}}\,{\text{insulin}}} \right)\left( {{\text{mean}}\,{\text{glucose}} \times {\text{mean}}\,{\text{insulin}}} \right)} }} $$

5 $$ McAuley Index = e^{(2.63 - (0.28\ln \,insulin) - (28.35\ln \,triglycerides)} $$

### Gene expression analysis

Trizol reagent (TransGen Biotech Co., China) was used to extract total RNA from the hepatic tissues and reverse transcriptase-polymerase chain reaction (RT-PCR) was used to analyse the expression of insulin receptor substrate 1 (*IRS-1*), glucose transporter isoform 4 (*GLUT-4*), *DPP-IV*, glucagon-like peptide-1 (*GLP-1*), protein kinase B (*AKT*) and phosphatidylinositol 3-kinase (*PI3K)* following set parameters and gene-specific primers in Table 1 for first-strand cDNA synthesis, with glyceraldehyde-3-phosphate dehydrogenase (*GAPDH*) as reference gene^21^. PCR products were run on ethidium bromide-stained agarose gel (1.5%) (Sigma Aldrich, Germany) in triplicates and viewed under UVP BioDoc-It Imaging system (Upland, CA, USA).

**Table 1:** Gene-specific primer sequence and RT-PCR parameter.

Gene	Primer sequence (5′–3′)	AT (°C)
*IRS-1*	TCCCCCTGCCCAAGGATATT (F) GAAAGGCAGTGGGTCTAGGG (R)	58
*GLUT-4*	CAACGTGGCTGGGTAGGCA (F) ACAACATCAGCCCAGCCGGT (R)	58
*DPP-IV*	GAGCTAATAACACCACTGAGGCAT (F) GAGCAAAAGTAGTACGTGCCAAG (R)	53
*GLP-1R*	CATCCACCTGAACC5GTTTGC (F) GGGCAGCGTCTTTGATGAA (R)	56
*AKT*	CCGCTATTATGCCATGAAGAT (F) TGTGGGCGACTTCATCCT (R)	54
*PI3K*	CAAAGCCGAGAACCTATTGC (F) GGTGGCAGTCTTGTTGATGA (R)	54
*GAPDH*	CTGACATGCCGCCTGAAAC (F) CCAGCATCAAAGGTGGAAGAA (R)	51

AT, annealing temperature.

### Pancreatic histology

The excised pancreatic tissues were histologically assessed as previously described^25,28^. Graded concentration of ethanol was used to dehydrate the excised pancreas fixed in forrmalsaline. The pancreas were then cleaned and cleared using xylene, impregnated and embedded in parraffin wax, mounted on microscopic slides, sectioned to about 5 μm and stained using haematoxylin and eosin (H&E). Slides were observed using a slide scanner (Leica SCN 4000, Leica Biosystems, Germany) and interpreted.

### Statistical analysis

Data are expressed as means ± S.E.M. Significant differences between multiple groups were determined using Duncan's multiple range test. *p* < 0.05 was considered as the significant value. All analyses were conducted using IBM SPSS v25 (IBM Inc., New York, USA) and Microsoft Excel 2019 (Microsoft Corporation, Redmond, Washington, USA) software packages.

## Results

### TCA treatment restores animal weight loss, plasma glucose level and clearance

**Figure 2:**
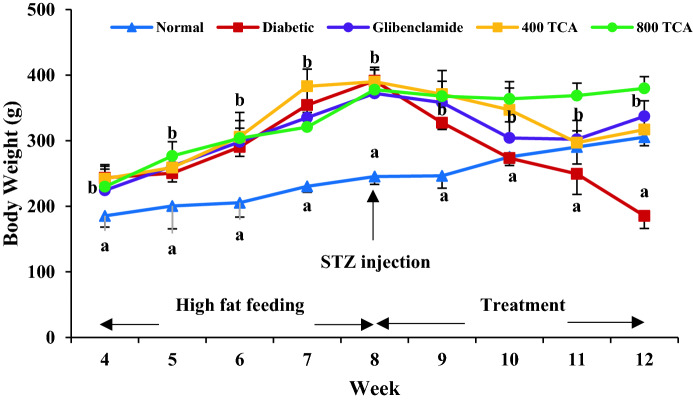
Effect of *T. catappa* aqueous extract treatment on HFD/STZ-induced diabetic rats body weight changes during the experimental period. Markers were expressed as mean ± SEM (n = 6). Markers with different superscripts for a given week are significantly different at *p* < 0.05. Markers without superscripts for a given week are significantly different at *p* < 0.05.

**Figure 3:**
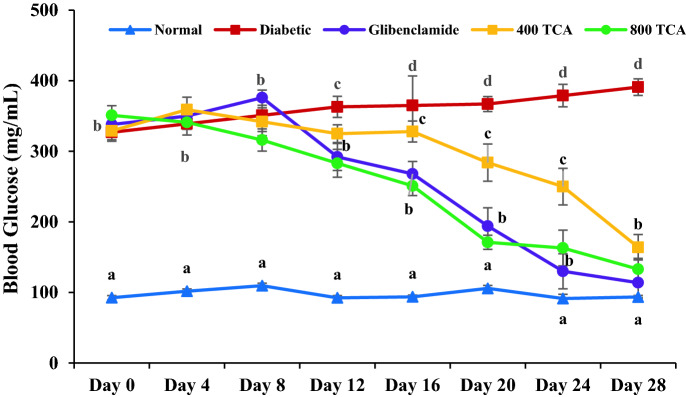
Effect of *T. catappa* aqueous extract treatment on HFD/STZ-induced diabetic rats’ mean fasting blood glucose changes during the experimental period. Markers are expressed as mean ± SEM (n = 6). Markers with different superscripts for a given day are significantly different at *p* < 0.05. Markers with similar superscripts for a given day are not significantly different at *p* < 0.05.

**Figure 4:**
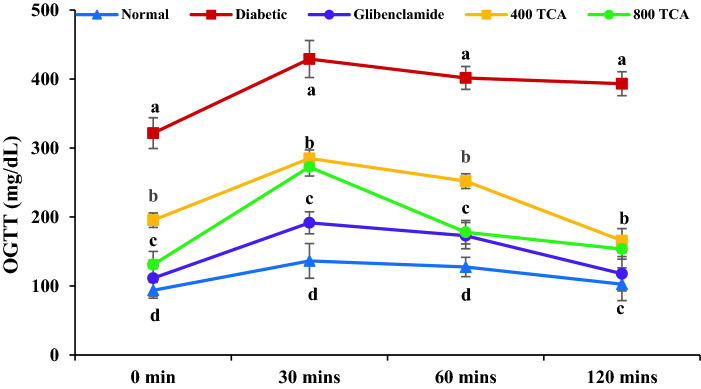
Effect of *T. catappa* aqueous extract treatment on HFD/STZ-induced diabetic rats’ oral glucose tolerance test. Markers are expressed as mean ± SEM (n = 6). Markers with different superscripts per time are significantly different at *p* < 0.05. Markers with similar superscripts per time are not significantly different at *p* < 0.05.

Rats fed HFD significantly (*p* < 0.05) gained more weight than those fed standard rat chow for the first eight weeks. Induction of diabetes using STZ led to significant (*p* < 0.05) weight loss in HFD/STZ-induced diabetic rats but administration of TCA significantly (*p* < 0.05) reduced the weight loss in a dose-dependent manner that was significant (*p* < 0.05) than glibenclamide at the highest dose (Fig. 2). During the intervention period, glibenclamide and TCA treatment groups showed a significant (*p* < 0.05) dose dependent decrease in blood glucose from the 12th day up until the end of the experimental regime compared with the untreated diabetic rats. The highest dose (800 mg/kg) of TCA decreased blood sugar at a rate which was comparable with glibenclamide (Fig. 3). In Fig. 4, induction of diabetes significantly affected (*p* < 0.05) the glucose tolerance of the untreated diabetic group compared with the normal group. In contrast, glibenclamide and TCA administration significantly (*p* < 0.05) improved glucose tolerance compared with untreated rats. The glucose tolerance of diabetic rats treated with the highest dose (800 mg/kg) of TCA and glibenclamide were significantly comparable to each other at 60 and 120 min.

### TCA treatment reinstates electrolyte imbalance

A significant (*p* < 0.05) increase in plasma urea (66.2%) and BUN (66.2%) concentration of rats induced with diabetes was observed compared with normal rats. Upon treatment, plasma urea and BUN concentrations of the diabetic rats were significantly (*p* < 0.05) reduced to normal at the TCA treated doses of 400 (27.8 and 26.9% respectively) and 800 (27.8 and 26.9% respectively) and glibenclamide (32.67 and 32.64% respectively) when compared with the diabetic and normal groups. The inverse was the case for plasma Ca, Na and HCO_3_ concentrations as they were significantly (*p* < 0.05) reduced in diabetic rats by 25.0, 39.2 and 50.9%, respectively. TCA treatment at the highest concentration significantly (*p* < 0.05) improved plasma Ca, Na and HCO_3_ concentrations to near normal levels by 15.4, 27.0 and 42.2% respectively when compared with diabetic group. The improvement of plasma Ca, Na and HCO_3_ concentrations by TCA was comparable with the improvement obtained in glibenclamide (13.51, 15.5 and 44.33% respectively) treated group. Despite these observations, induction of diabetes and TCA treatment had no significant (*p* > 0.05) effect on plasma Crea level in comparison with normal rats (Table 2).

**Table 2:** Effect of T. catappa aqueous extract treatment on kidney function parameters in HFD/STZ-induced diabetic rats.

	Normal	Diabetic	Glibenclamide	TCA 400	TCA 800
Urea (mg/dL)	22.94 ± 1.23^a^	38.12 ± 1.59^b^	25.66 ± 0.63^a^	27.53 ± 2.14^a^	27.87 ± 1.51^a^
Ca (mg/dL)	9.86 ± 1.10^c^	7.40 ± 0.25^a^	8.40 ± 2.47^b^	8.55 ± 2.51^b^	8.54 ± 1.24^b^
Na (mEQ/L)	92.12 ± 18.20^c^	56.03 ± 20.51^a^	64.52 ± 21.13^a^	52.71 ± 16.61^a^	71.17 ± 18.27^b^
BUN (mg/dL)	10.71 ± 0.57^a^	17.80 ± 0.74^b^	11.99 ± 0.29^a^	12.85 ± 1.00^a^	13.01 ± 0.70^a^
HCO_3_ (mmol/L)	28.02 ± 8.03^c^	13.76 ± 6.71^a^	19.86 ± 2.31^b^	20.13 ± 1.06^b^	19.57 ± 1.22^b^
Crea (mg/dL)	0.58 ± 0.17	0.95 ± 0.02	0.62 ± 0.08	0.70 ± 0.12	0.61 ± 0.05

Data are represented as mean ± SEM (n = 6). Values with different superscripts across a row are significantly different at *p* < 0.05. Values without superscripts across a row are not significantly different at *p* < 0.05. TCA: *T. catappa* aqueous extract ALT: alanine transaminase, AST: aspartate aminotransferase, ALP: alkaline phosphatase, ALB: albumin, BIL: bilirubin, Ca: calcium, Na: sodium, BUN: blood urea nitrogen, HCO_3_: bicarbonate and Crea: creatinine.

### TCA treatment palliates dysregulated diabetic parameters

**Figure 5:**
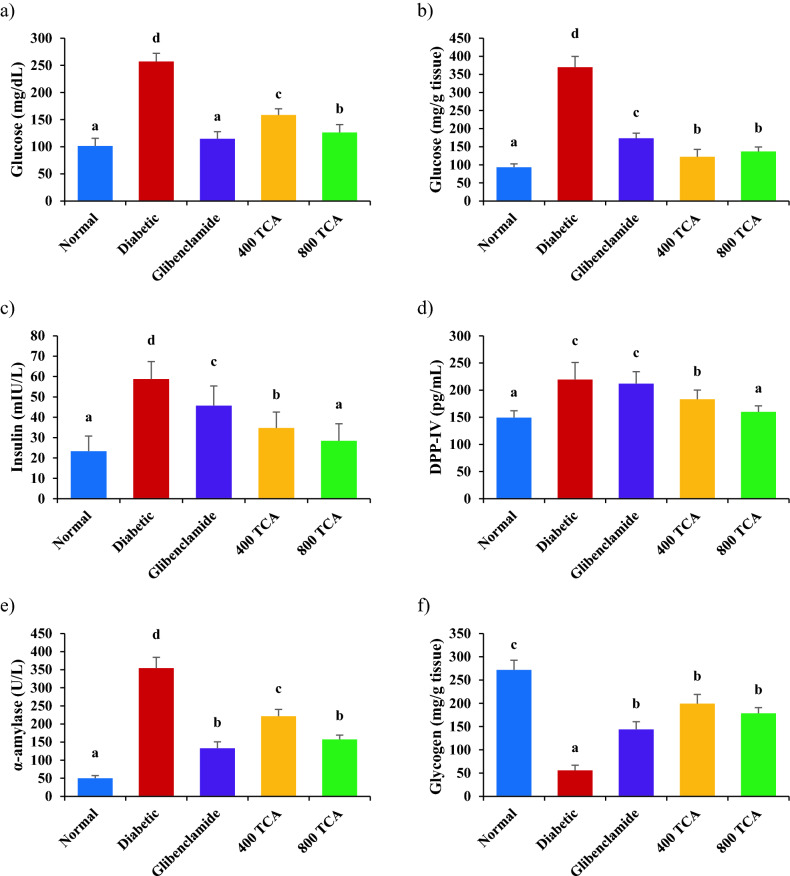

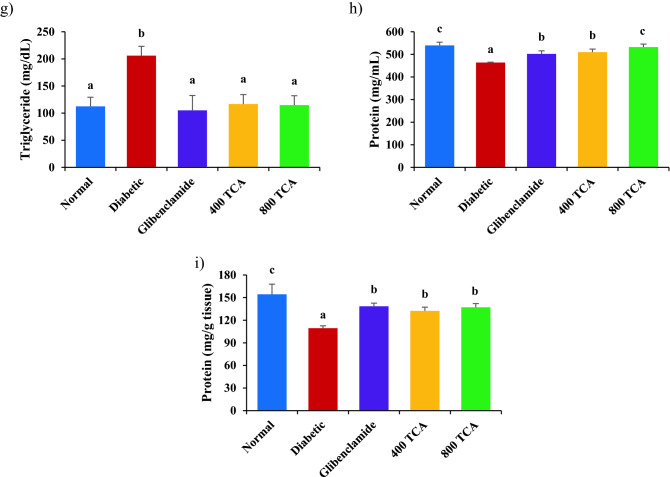
Effect of *T. catappa* aqueous extract treatment on (**a**) plasma glucose, (**b**) liver glucose, (**c**) plasma insulin, (**d**) plasma dipeptidyl peptidase IV, (**e**) plasma α-amylase, (**f**) hepatic glycogen, (**g**) triglyceride, (**h**) plasma protein and (**i**) hepatic protein in HFD/STZ-induced diabetic rats. Bars are expressed as mean ± SEM (n = 6). Bars with different superscripts are significantly different, while those with similar superscripts are not significantly different at *p* < 0.05.

Induction of diabetes resulted in a significant (*p* < 0.05) increase in plasma GLUC, liver GLUC, INS, DPP-IV, AMY and TRIG by 153.5, 296.0, 152.0, 47.0, 610.0 and 83.2%, respectively, in diabetic rats compared with normal rats. Glibenclamide and TCA treatment at 400 and 800 mg/kg bw, significantly (*p* < 0.05) decreased plasma GLUC (55.4, 38.3 and 50.9%, respectively), liver GLUC (53.1, 66.9 and 63.0%, respectively), INS (22.2, 40.9 and 51.6%, respectively), DPP-IV (16.6 and 27.1%, respectively), AMY (62.5, 37.6 and 55.6%, respectively) and TRIG (49.0, 43.3 and 44.3%, respectively) in diabetic treated rats compared with untreated diabetic rats. It was noteworthy that the highest dose of TCA significantly reduced plasma insulin in diabetic rats to normal levels compared with normal rats while glibenclamide did not have a significant effect on DPP-IV in diabetic rats (Fig. 5). The inverse was the case for GLYC (79.5%), plasma TP (14.1%) and hepatic TP (29.2%) concentrations in diabetic rats as they were significantly (*p* < 0.05) depleted after induction of diabetes compared with normal rats. However, upon treatment with glibenclamide, 400 and 800 mg/kg bw TCA, GLYC (157.8, 257.0 and 219.4%, respectively), plasma TP (8.3, 10.0 and 14.8%, respectively) and hepatic TP (26.6, 21.0 and 25.4%, respectively) concentrations of the diabetic treated rats were significantly (*p* < 0.05) improved compared to the untreated diabetic rats (Fig. 5).

### TCA treatment improves β-cell function and insulin resistance indicators

**Figure 6:**
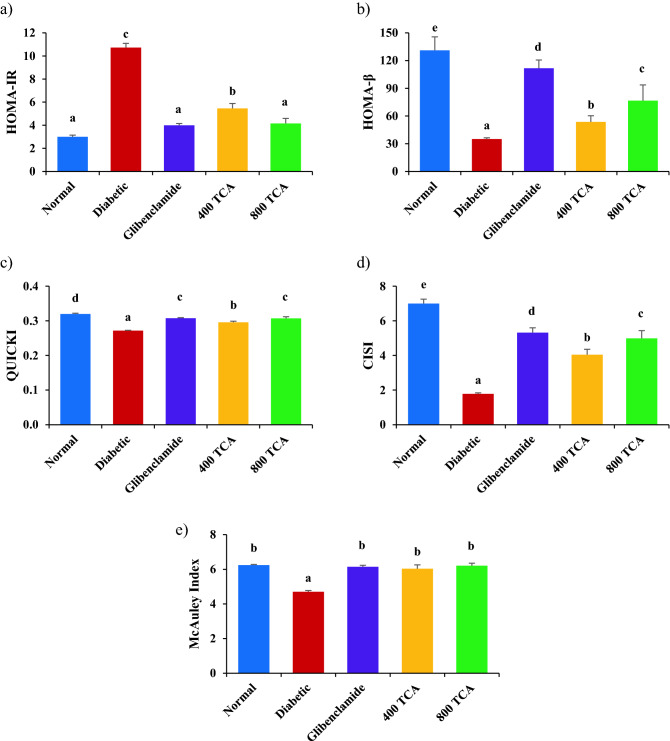
Effect of *T. catappa* aqueous extract treatment on (**a**) homeostasis model assessment for insulin resistance, (**b**) homeostasis model assessment for β-cell function, (**c**) quantitative insulin-sensitivity check index, (**d**) composite insulin sensitivity index and (**e**) McAuley index in HFD/STZ-induced diabetic rat. Bars are expressed as mean ± SEM (n = 6). Bars with different superscripts are significantly different, while those with similar superscripts are not significantly different at *p* < 0.05.

Diabetes-related indices HOMA-β, QUICKI, CISI and MI in Fig. 6 were significantly (*p* < 0.05) reduced in diabetic rats by 73.3, 15.1, 74.5 and 24.6%, respectively, compared with normal rats. Diabetic rats treated with glibenclamide, 400 and 800 mg/kg bw TCA showed significant (*p* < 0.05) improvement for HOMA-β (219.1, 53.3 and 118.8%, respectively), QUICKI (13.2, 8.9 and 13.1%, respectively) and CISI (197.9, 126.6 and 179.4%, respectively) in a dose-dependent pattern while for MI (30.6, 28.2 and 32.0%, respectively), all treatments significantly (*p* < 0.05) restored this index to normal level when compared with untreated diabetic and normal rats. For HOMA-IR, induction of diabetes significantly (*p* < 0.05) increased this index by 257.6% compared with normal rats. Diabetic rats treated with glibenclamide, 400 and 800 mg/kg bw TCA showed a significant (*p* < 0.05) decrease by 62.8, 49.1 and 61.3%, respectively, for this index (Fig. 6).

### TCA treatment reverses disrupted mRNA expression

**Figure 7:**
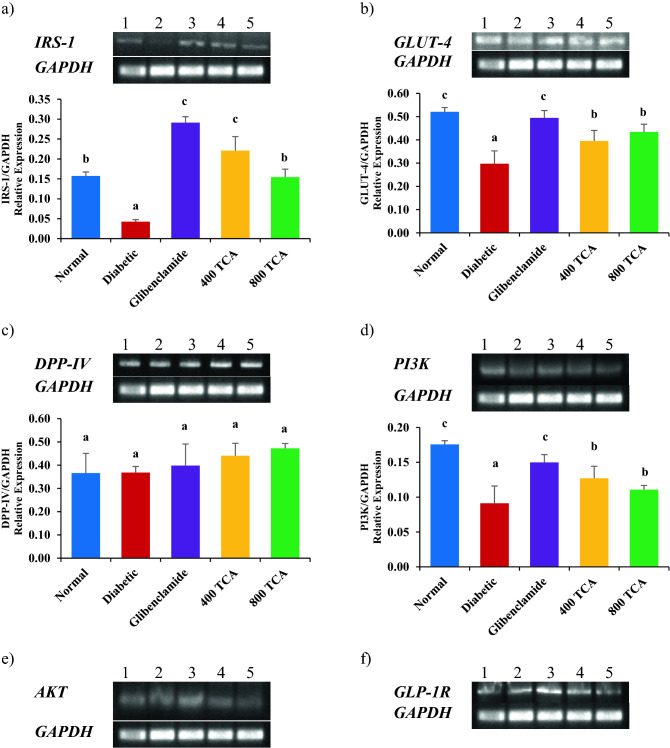

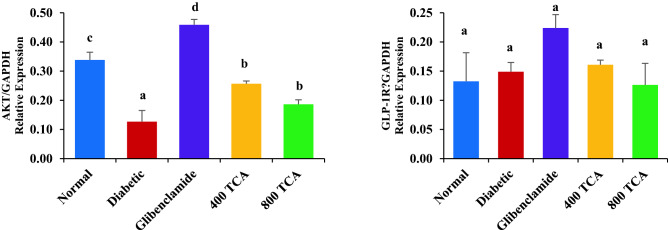
Effect of *T. catappa* aqueous extract treatment on hepatic (**a**) *IRS-1*, (**b**) *GLUT-4*, (**c**) *DPP-IV*, (**d**) *PI3K*, (**e**) *AKT* and (**f**) *GLP-1R* mRNA expression in HFD/STZ-induced diabetic rat. Bars are expressed as mean ± SEM (n = 6). Bars with different superscripts are significantly different, while those with similar superscripts are not significantly different at *p* < 0.05.

Induction of diabetes significantly (*p* < 0.05) reduced the expression of hepatic *IRS-1, GLUT-4, P13K* and *AKT* mRNA in the diabetic rats compared with normal rats. Treatment of diabetic rats with glibenclamide and TCA significantly (*p* < 0.05) improved the expression of these genes in the experimental group compared with the untreated diabetic animals. However, there was no significant (*p* > 0.05) change in *DPP-IV* mRNA expression even after the onset of diabetes and subsequent glibenclamide and TCA treatment (Fig. 7 and S1).

### TCA treatment repairs damaged pancreatic histology

**Figure 8:**
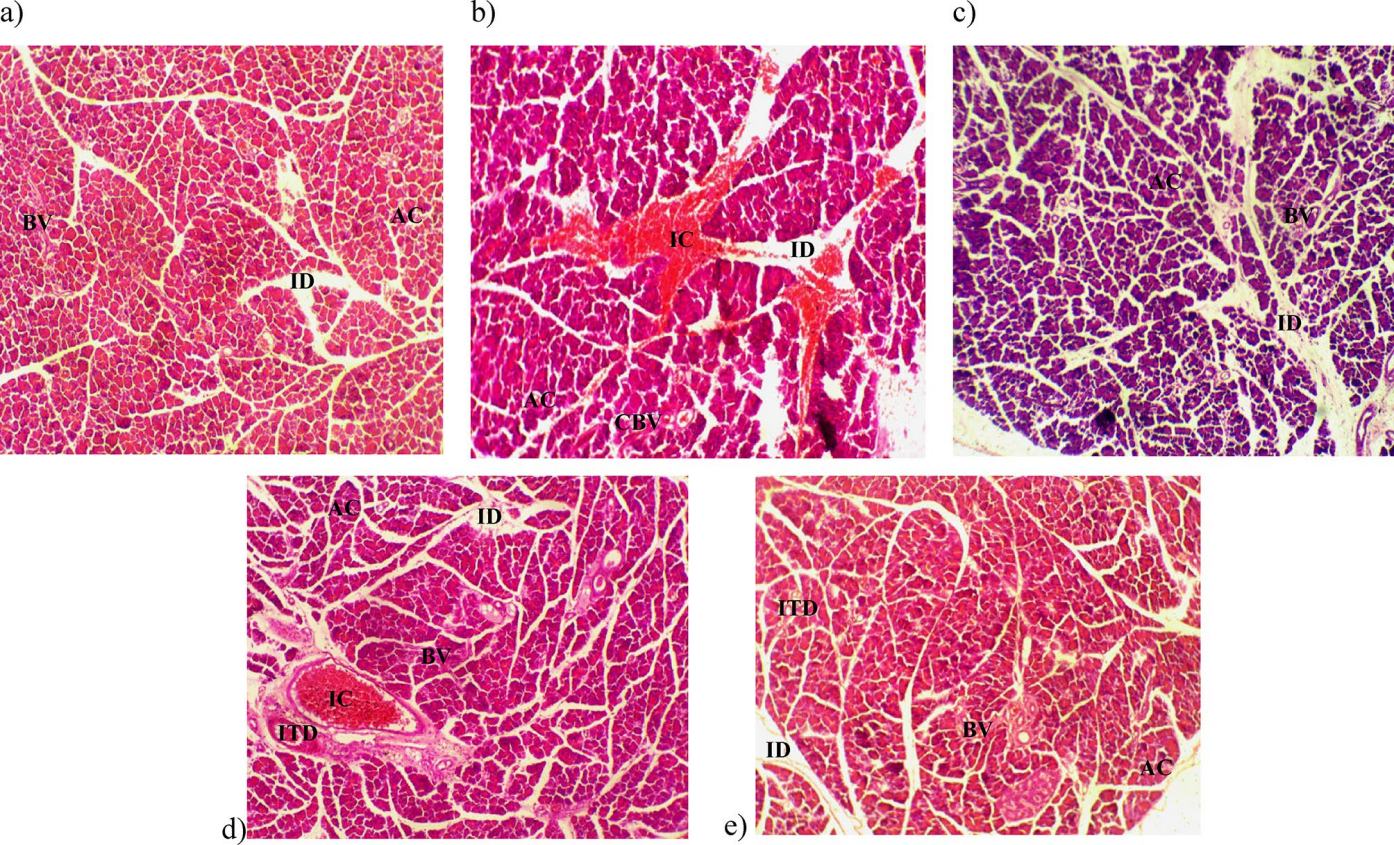
Pancreatic histology of (**a**) normal (**b**) diabetic (**c**) glibenclamide (**d**) 400 mg/kg bw and (**e**) 800 mg/kg bw *T. catappa* aqueous extract treatment of HFD/STZ-induced diabetic rats (H&E × 100). ID: intralobular duct, BV: blood vessel, AC: acini, IC: inflammatory cell, CBV: congested blood vessel, ITD: interlobular duct.

Histopathology of pancreatic tissues revealed distinct exocrine acini with islets, blood vessel intralobular and interlobular duct in normal rats, glibenclamide and TCA 800 mg/kg bw treated rats. For the untreated diabetic rats, vascular congestion significant with pancreatitis, acini and intralobular duct were aggregated with inflammatory cells and blood vessels congested with red blood cells were observed. In the pancreatic tissues of 400 mg/kg bw TCA-treated rats, a moderate aggregate of inflammatory cells were observed in the intralobular and interlobular duct (Fig. 8).

## Discussion

Rats that have diabetes induced by STZ exhibit austere body weight loss. This observation has been attributed to the rapid catabolism of structural proteins due to carbohydrates being unavailable to be utilised as a viable energy source^35^. This study further corroborates previous reports as treatment with STZ led to a loss of body weight in diabetic rats^36,37^. The reversal of this anomaly upon treatment with TCA suggests the protective property of the extract and its phytoconstituents on structural proteins, thereby preventing their degradation. OGTT is a more dependable assessment of detecting early glucose homeostasis abnormalities than fasting blood glucose as it depicts blood glucose uptake and hepatic gluconeogenesis after a glucose loaded/rich meal^38^. Excessive time-dependent elevation of blood glucose level indicated impaired glucose tolerance. The reduction observed after extract treatment indicated the mitigating effect of TCA on impaired glucose tolerance, further confirming the ability of the extract to improve insulin sensitivity in the tissues^38^. TCA also reduced the blood glucose levels monitored daily, suggesting glucose homeostasis was being restored to normal. This finding was similar to previous works where *Xylopia aethiopica* and *Aframomum melegueta* fruits were used^31,39^.

The observed increase in plasma urea, BUN and decrease in plasma Na, Ca and HCO_3_ after induction of diabetes is similar to the report of Ekperikpe et al.^40^. Similarly, the damage to the kidney, as observed in diabetics, is characterised by distortions in electrolyte homeostasis, giving rise to increased metabolites excretion above normal thresholds^41^. The induced loss of serum Na^+^ and K^+^ levels during diabetes onset is majorly due to dehydration^42^. Diabetic ketoacidosis is also a condition that arises due to the inability to utilize glucose for energy production hence generating ketone bodies and elevating acidosis increasing serum Cl^-^ while reducing HCO_3_^-^ levels. The diabetic condition brings about a derangement in glucose production pathways leading to blood acidification, causing acid–base derangement with a subsequent increase in Cl^-^ level to balance it^36^. Inhibition of the renin–angiotensin–aldosterone system as experienced in many endocrine systems may also play a role in electrolyte imbalance. Increase in plasma urea concentration is a known hallmark of diabetes nephropathy, as similarly observed in this study^43^. TCA administration modulated these observed electrolyte imbalances, suggesting it can restore tubular function in the kidney of diabetic rats and electrolyte homeostasis by attenuating diabetes-related hyponatremia, ketoacidosis and osmotic diuresis^44^. The quick attenuation of ketoacidosis would suggest a T2DM-related ketoacidosis as its resolution is significantly shorter than in T1DM^45^. The observed T2DM-associated ketoacidosis in our study was similar to previous studies where IR leads to increased HCO_3_^-^ levels and plasma α-amylase activity^46,47^.

The ability of the body to stimulate the uptake, utilise and store glucose through the action of insulin is a critical factor in maintaining a healthy blood glucose level^48,49^. The observed rise in blood and hepatic glucose with a concomitant rise in plasma insulin concentration, plasma α-amylase and DPP-IV activities in diabetic rats was consistent with the findings of other researchers who used the HFD and low dose STZ diabetes induction model^26,46,50–52^. Interestingly, some other studies have reported insulin levels to be lower in the HFD/low dose STZ diabetes induction model despite the spike in blood glucose levels^53–56^. Diet manipulation to increase body fat content (HFD) combined with low dose administration of STZ induces insulin resistance and significantly elevates blood glucose levels^56^. The excessive intake of diet rich in fat also accelerates the release of insulin from the β–cell of the pancreas^46^. It is a model used to conveniently induce both hyperglycaemia and metabolic profiles in rats exhibiting close similarity to the clinical progression of T2DM in humans^57^. Protein and glycogen storage impairment have been reported to have a positive association with diabetes and an increase in hepatic glucose concentration. TCA's reversal of these abnormal conditions could be attributed to its ability to increase tissues insulin sensitivity thereby increasing the ability of the organs to utilise glucose for energy generation, in the process reducing plasma glucose concentration, insulin concentration, α-amylase and DPP-IV activity^58^. Reduced hepatic glycogen synthesis in the diabetic state has been attributed to irregular catabolism of hepatic glycogen stores and impairment of signals that stimulate hepatic glucose uptake and storage^30^. Plasma and organ protein levels are affected when there is insufficient glucose utilisation as the main source of energy generation^59^. The utilisation of glucose by the tissues will lead to increased generation of ATP, which may be responsible for the anabolic effect of insulin on protein metabolism retarding the observed reduction of plasma and organ protein concentration as well as the restoration of normal synthesis and storage of glycogen in the liver^60^.

The restoration of glucose homeostasis to normal may be attributed to the upregulation in *IRS-1, GLUT-4, PI3K* and *AKT* mRNA expression in the liver, which was reduced during the diabetes onset^61^. In normal physiology, insulin binding to the insulin receptor leads to its phosphorylation and initiation of insulin action. Activation of IRS-1, PI3K and AKT in a sequential manner leads to GLUT-4 vesicle translocation to the cell membrane, which finally induces glucose uptake in tissues as the concomitant effect of these chains of metabolic processes^12^. During T2DM onset, signal transduction of IRS-1/PI3K/Akt pathway is downregulated, attenuating GLUT-4 vesicles' translocation, leading to the inability of tissues to take up glucose^62^. The induction of IRS-1/PI3K/Akt pathway upregulation concomitantly enhancing hepatic glucose uptake may be the attributable molecular mechanism of TCA in improving overall glucose homeostasis and overcoming insulin resistance in diabetes^63^. The molecular mechanism of action of TCA on HFD/low dose STZ diabetic rat model was first reported by our team.

These biochemical observations were further corroborated with the results for indices of β–cell function and insulin resistance. Induction of diabetes reduced QUICKI, CISI, HOMA-β and McAuley indices in the rats with an increase in HOMA-IR as a consequence. TCA treatment attenuated these abnormal values, suggesting TCA's antidiabetic action could be attributed to the reversal of insulin resistance, partial pancreatic beta-cell dysfunction, and decreased insulin sensitivity. Mohammed et al.^39^ and Mudi et al.^64^ had the same findings on HFD/STZ induced T2DM rats model using *Xylopia aethiopica* fruit acetone fraction and *Aegle marmelos* fruit and leaf aqueous extract, respectively, thus corroborating the study. These indices are used to precisely determine insulin sensitivity, resistance, β-cell function and glucose‑insulin homeostasis, both clinically and epidemiologically^65^. The reversal of damaged pancreatic histology by TCA might suggest its ability to regenerate distorted pancreatic tissues.

**Figure 9:**
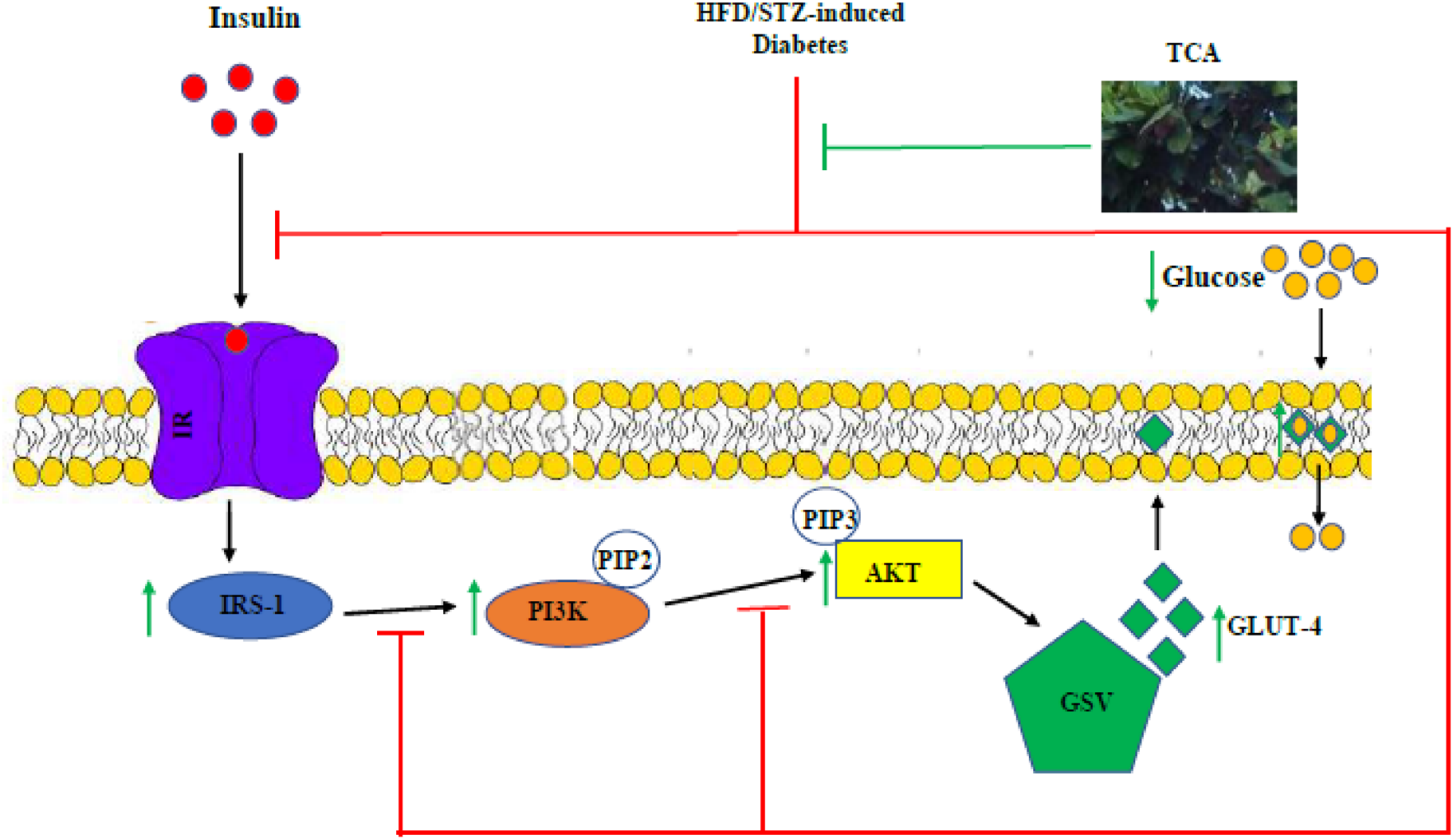
Proposed antidiabetic molecular mechanism of *T. catappa* aqueous extract in HFD/STZ-induced diabetic rats. IR: insulin receptor, IRS-1: insulin receptor substrate 1, PI3K: phosphatidylinositol 3-kinase, PIP2: phosphatidylinositol-4,5-bisphosphate, PIP3: phosphatidylinositol-3,4,5-bisphosphate, AKT: protein kinase B, GSV: GLUT-4 storage vesicle and GLUT-4: glucose transporter isoform 4.

In summary, this study shows that TCA ameliorates diabetes and its resultant downstream effect in high fat/low dose streptozotocin-induced diabetic rats to improve dysregulated glucose metabolism. The mechanisms at which these ameliorating effects are achieved may be via *GLUT-4, IRS-1, PI3K* and *AKT* gene upregulation which may probably enhance GLUT-4 translocation, reduce insulin resistance and induce PI3K/AKT signalling pathway (Fig. 9). Hence, TCA could be exploited for efficient antidiabetic agents to remedy diabetes and restore glucose metabolism. Inability to study the protein expression of evaluated genes to corroborate the gene expression result and other mechanisms through which TCA might have exerted its antidiabetic effect were some limitations of this study. In addition, random sampling of the hepatic tissue might have been a confounder in glycogen assessment. Therefore, novel mechanisms of TCA and further molecular assessment of protein expression should be studied to understand its antidiabetic action fully.

## Supplementary Information

Supplementary Figure S1.

## Data Availability

All data are included in the manuscript and supplementary file.
