# Supplementary material for: Terminalia catappa aqueous leaf extract reverses insulin resistance, improves glucose transport and activates PI3K/AKT signalling in high fat/streptozotocin-induced diabetic rats
Source: Sci Rep. 2022 Jun 23;12:10711. doi: 10.1038/s41598-022-15114-9 (PMC9226017; doi:10.1038/s41598-022-15114-9)
Supplement: Supplementary file 1 — Supplementary Figure S1. [file 41598_2022_15114_MOESM1_ESM.docx]

**Terminalia catappa reverses insulin resistance, improves glucose transport and activates PI3K/AKT signalling in high fat/ streptozotocin-induced diabetic rats**

**Franklyn Nonso Iheagwam^1,2*^, Olawumi Toyin Iheagwam^3^, Michael Kemjika Onuoha^2,4^, Olubanke Olujoke Ogunlana^1,2^ and Shalom Nwodo Chinedu^1,2^**

^1^Department of Biochemistry, Covenant University, P.M.B. 1023 Ota, Ogun State, Nigeria.

^2^Covenant University Public Health and Wellbeing Research Cluster (CUPHWERC), Covenant University, P.M.B. 1023 Ota, Ogun State, Nigeria.

^3^Geniebook Associates, Ikeja, Lagos State, Nigeria.

^4^Covenant University Health Centre, Covenant University, P.M.B. 1023 Ota, Ogun State, Nigeria.

*Corresponding Author: [franklyn.iheagwam@covenantuniversity.edu.ng](mailto:franklyn.iheagwam@covenantuniversity.edu.ng)

a)
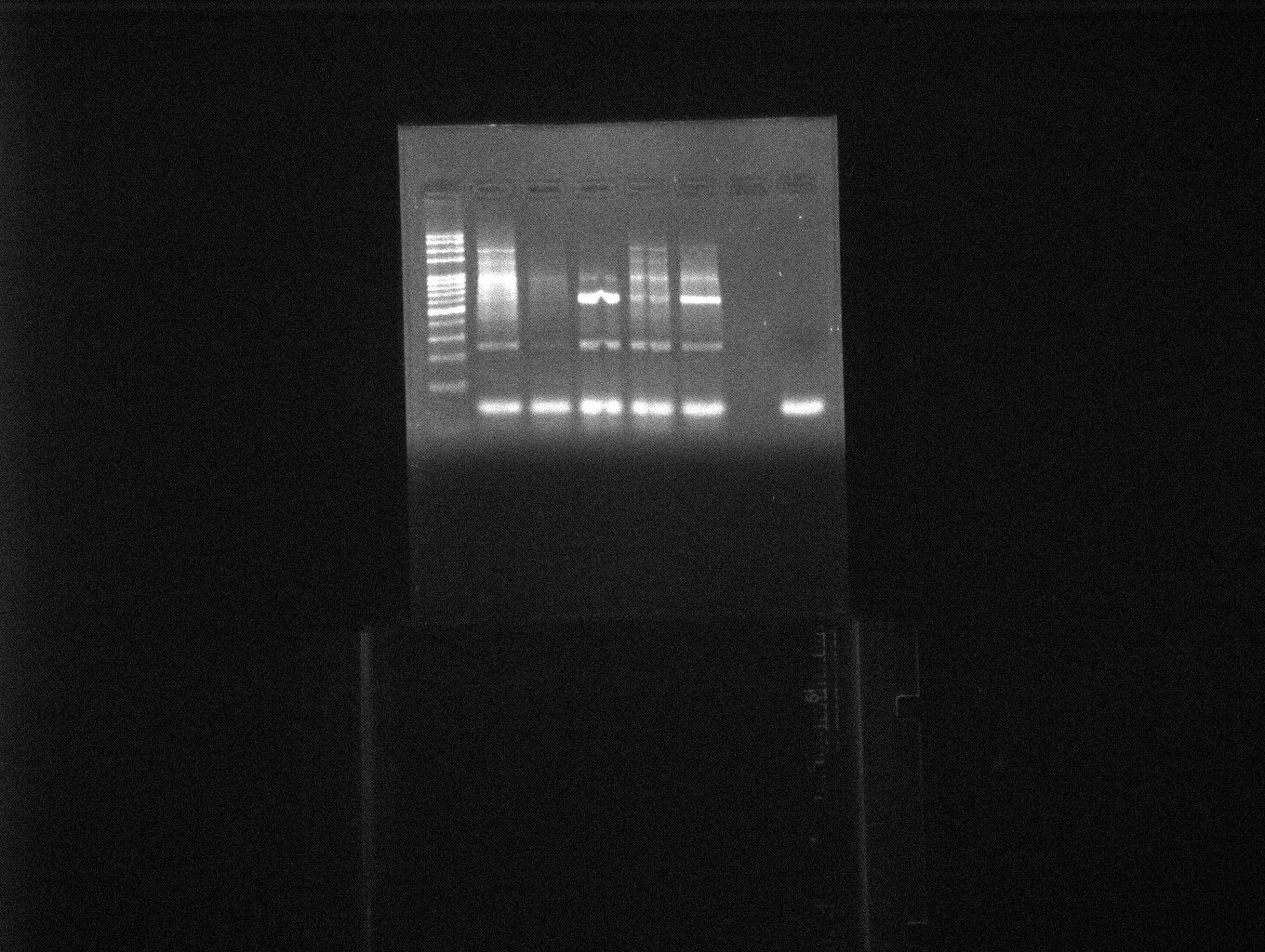


b)
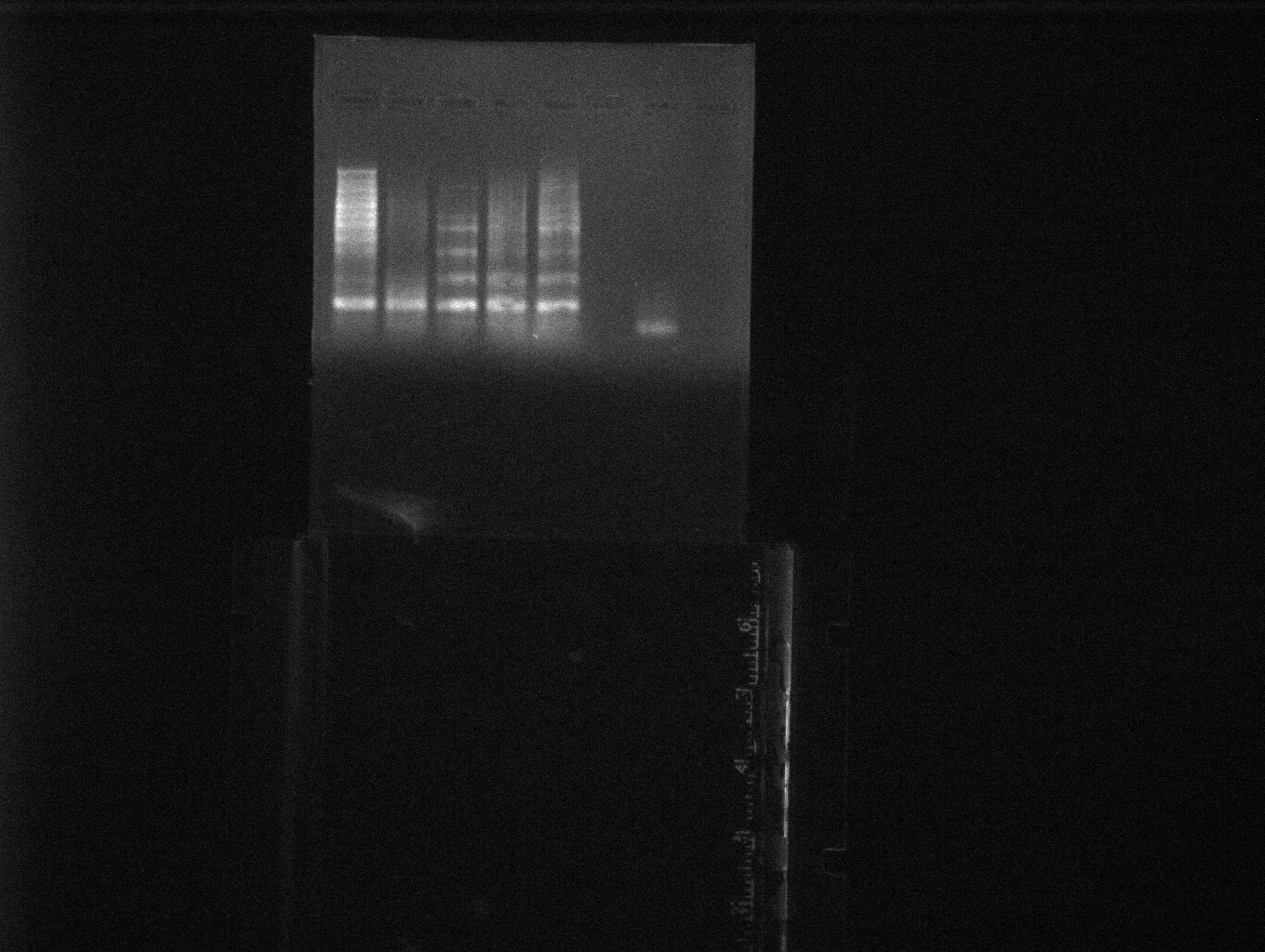


c)
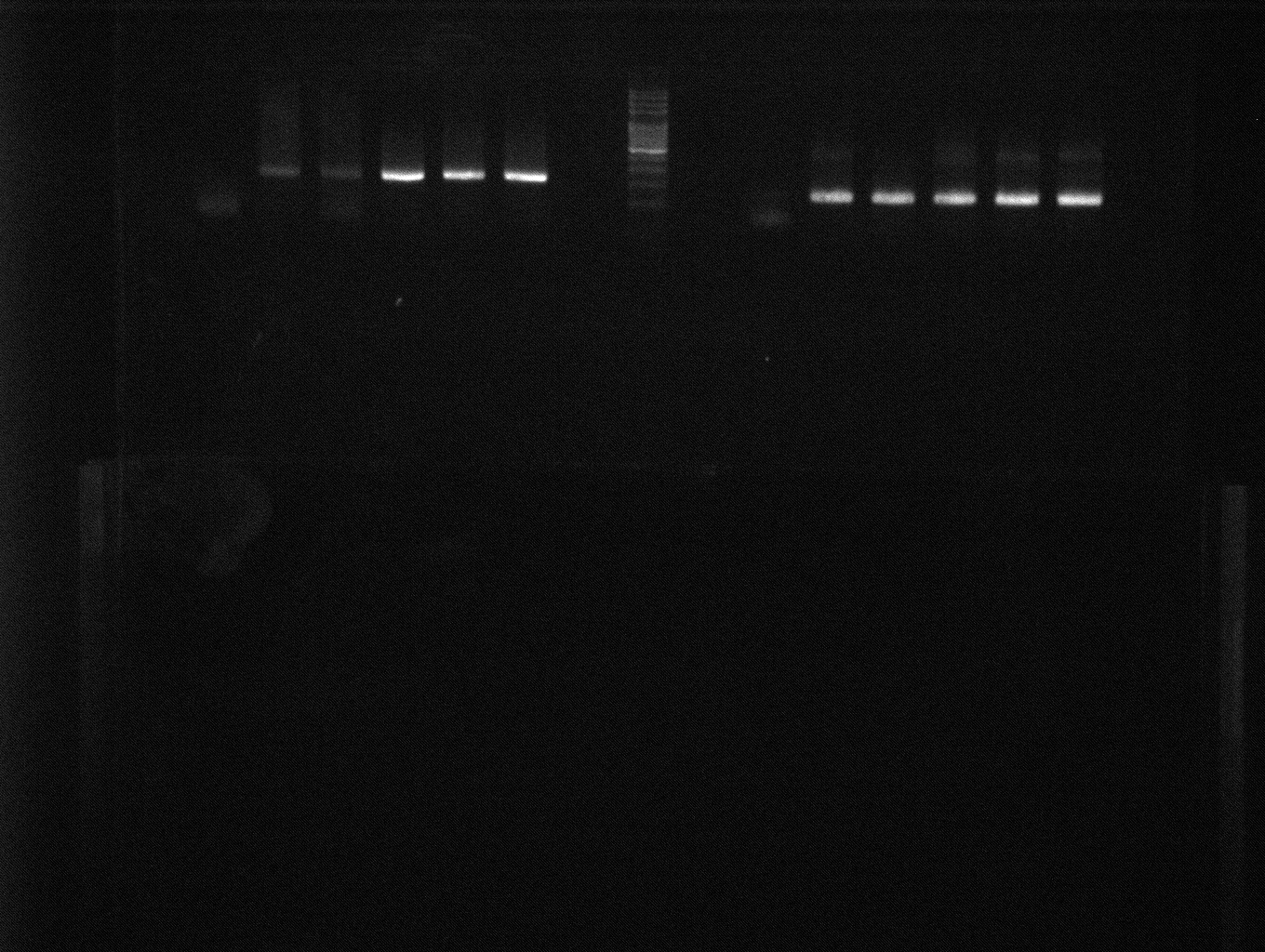


d)
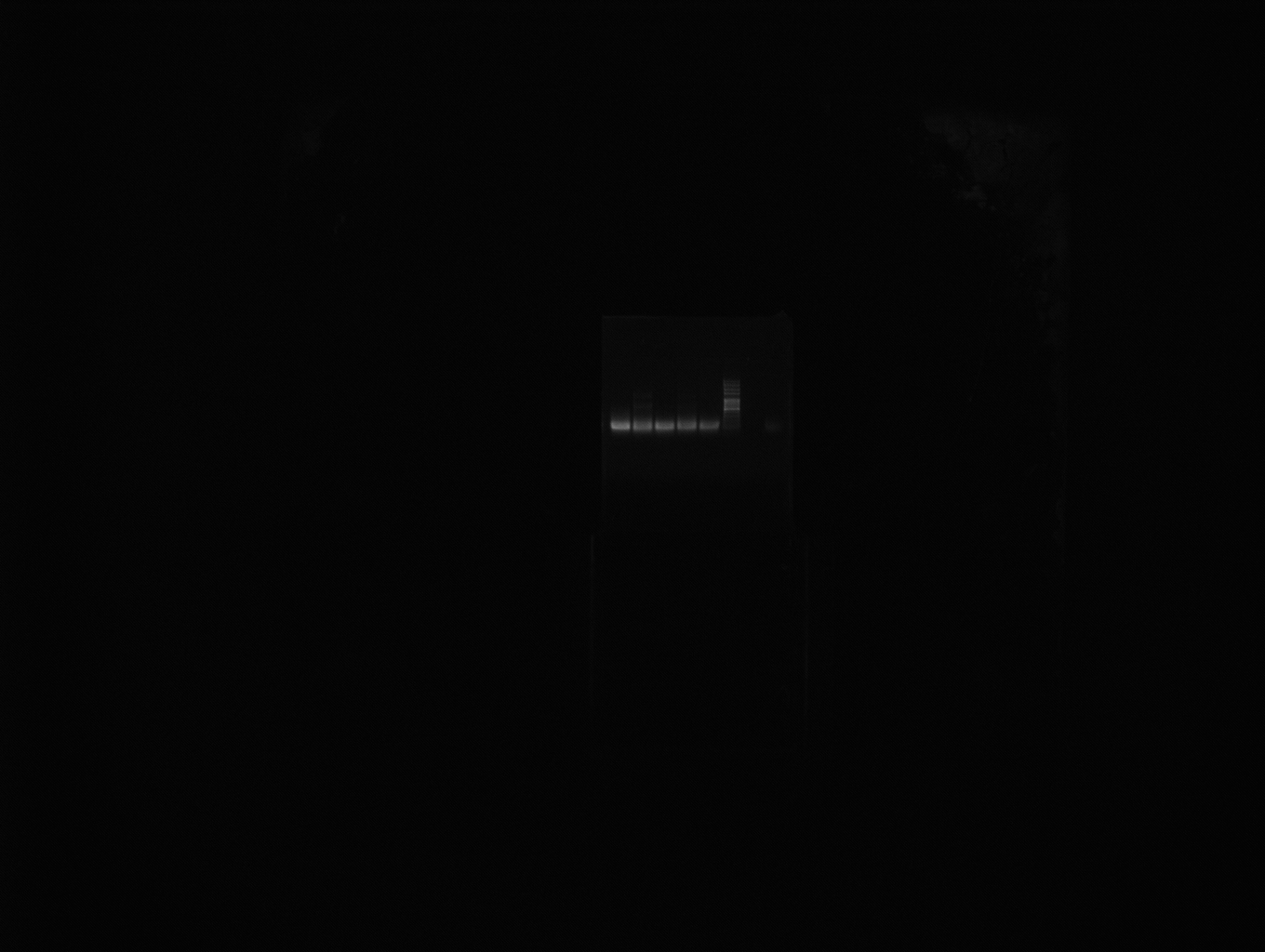


e)
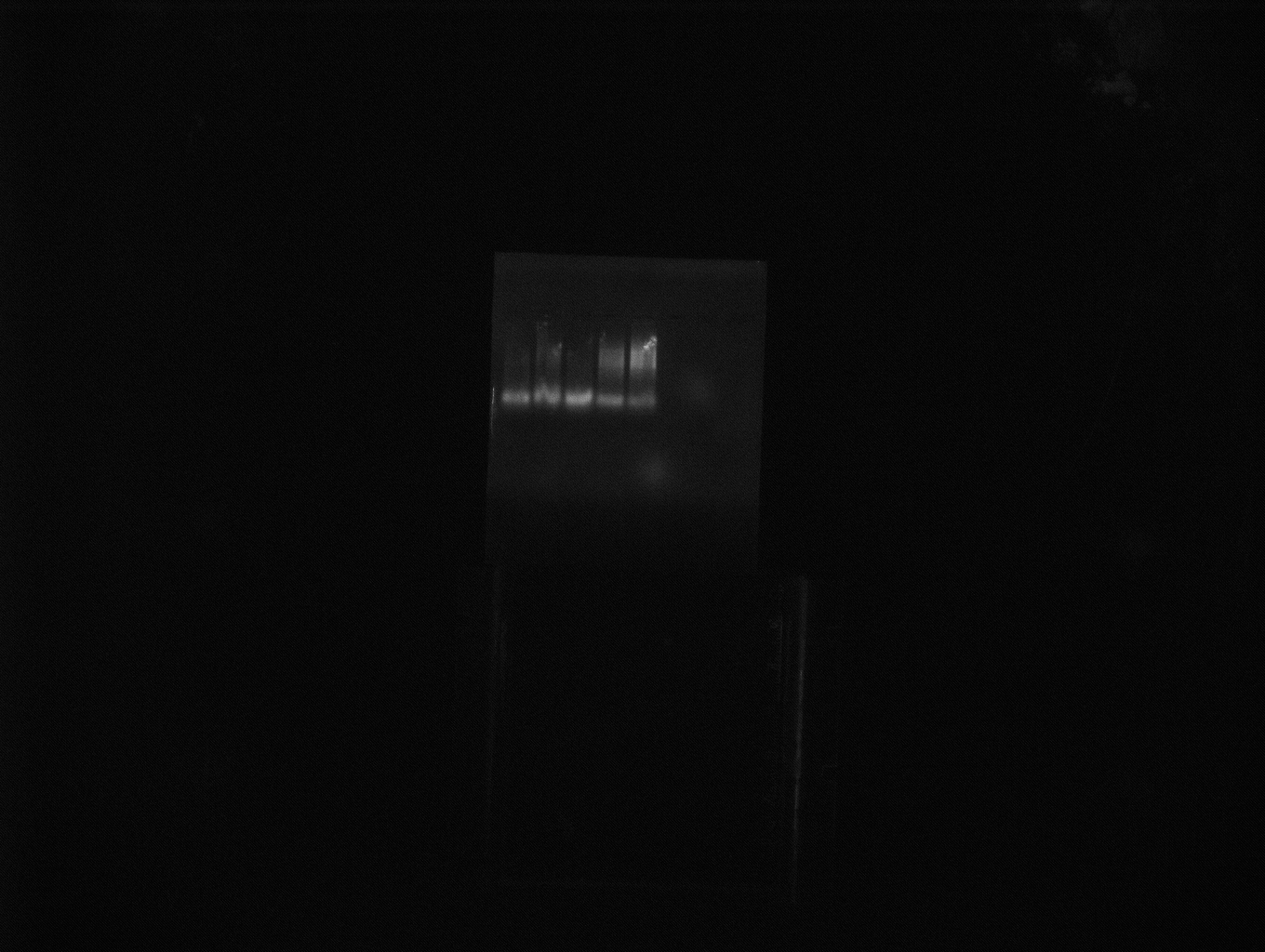


f)
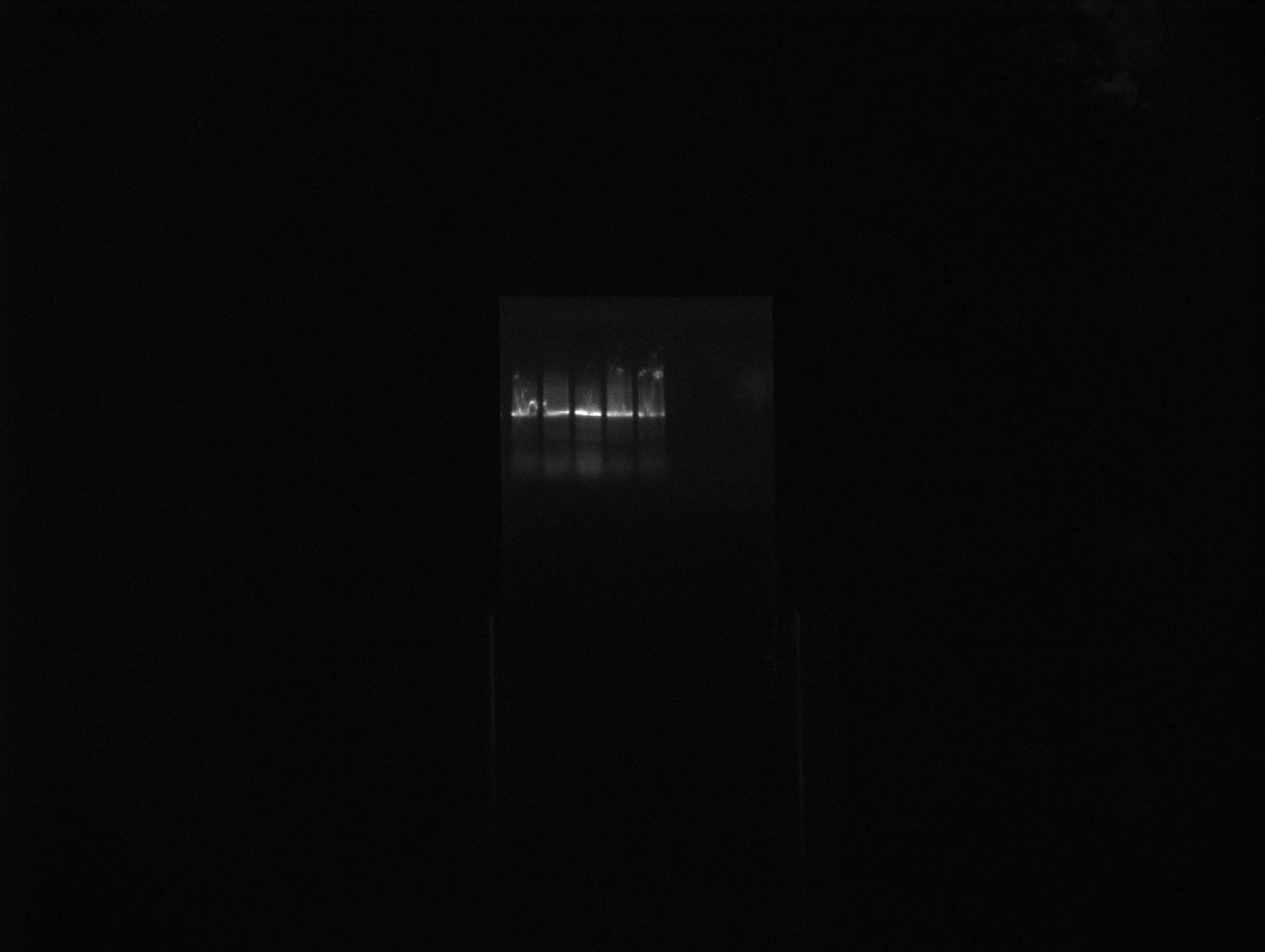


g)
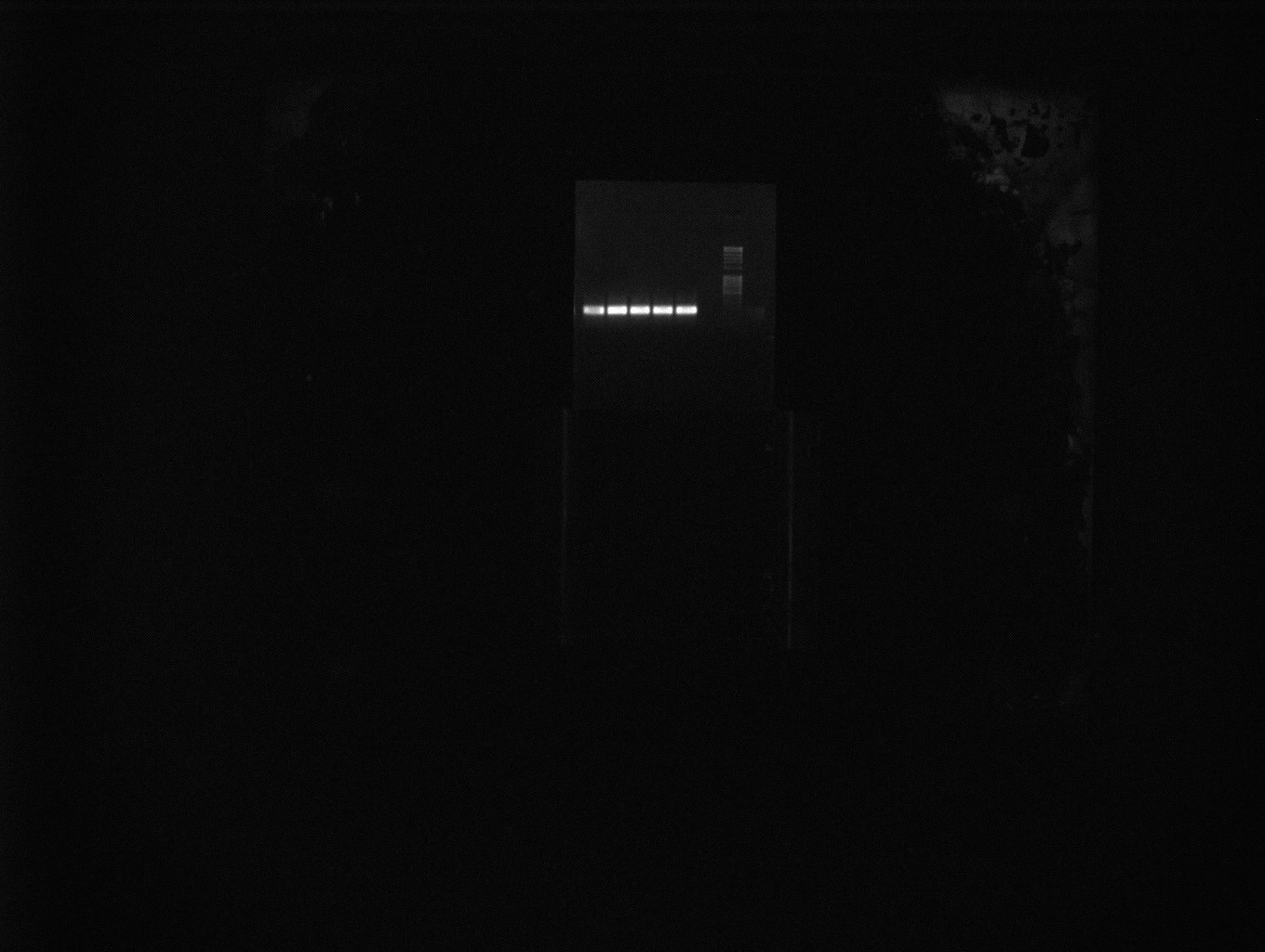


**Figure S1:** Supplementary figures for the original source of (a) *IRS-1*, (b) *GLUT-4*, (c) *DPP4*, (d) *PI3K*, (e) *AKT*, (f) *GLP* and (g) *GAPDH* gel images
